# Optimization of WEDM Parameters While Machining Biomedical Materials Using EDAS-PSO

**DOI:** 10.3390/ma16010114

**Published:** 2022-12-22

**Authors:** Vishal S. Sharma, Neeraj Sharma, Gurraj Singh, Munish Kumar Gupta, Gurminder Singh

**Affiliations:** 1 Department of Industrial and Production Engineering, Dr. B R Ambedkar National Institute of Technology, Jalandhar 144027, India; 2 School of Mechanical, Industrial and Aeronautical Engineering, University of Witwatersrand, Johannesburg 2000, South Africa; 3 Mechanical Engineering Department, Maharishi Markandeshwar (Deemed to Be University), Ambala 134003, India; 4 Faculty of Mechanical Engineering, Opole University of Technology, 76 Proszkowska St., 45-758 Opole, Poland; 5 Department of Mechanical Engineering, Indian Institute of Technology Bombay, Mumbai 400076, India

**Keywords:** EDAS-PSO, optimization, pure titanium (Grade 2), WEDM

## Abstract

In the present work, an attempt has been made to study the influence of process parameters of the wire electric discharge machining (WEDM) process on the machining characteristics. The commercially pure titanium is machined by WEDM using brass wire as an electrode. The input parameters in this work were pulse on-time (A_on_), pulse off-time (A_off_), servo voltage (SV) and wire tension (WT). On the other hand, dimensional accuracy (DA), average surface roughness (R_a_) and maximum surface roughness (R_z_) were chosen as the response parameters. The empirical relations developed for response characteristics were solved collectively using Evaluation Based on Distance from Average Solution (EDAS) and Particle Swarm Optimization (PSO). The optimized setting for minimizing the surface irregularities while machining titanium alloy on WEDM is predicted as A_on_: 8 μs; A_off_: 13 μs; SV: 45 V; and WT: 8 N. Moreover, the predicted solution at the optimized parametric settings came out as DA: 95%; Ra: 3.163 μm; Rz: 22.99 μm; WL: 0.0182 g; and DR: 0.1277 mm. The validation experiments at the optimized setting showed the close agreement between predicted and experimental values. The morphological study by scanning electron microscopy (SEM) at the optimized setting revealed a significant reduction in surface defects such as micro cracks, micro cavities, globules and sub-surfaces, etc. In a nutshell, the study justified the effectiveness of EDAS-PSO in efficiently predicting the results for machining of pure titanium (Grade 2) using the WEDM process.

## 1. Introduction

Titanium and its alloys are utilized in a wide range of industrial and biomedical applications. Their properties such as corrosion resistance at elevated temperatures and higher strength–density value place them among the most desirable and appropriate materials for such engineering applications. Moreover, their biocompatibility paves the way for their widespread usage as biomaterials [1,2,3]. Notwithstanding their advantages, these materials are also categorized as hard to machine materials owing to their high chemical reactivity as well as lower thermal conductivity [4,5]. Additionally, for their economic viability, the production rates as well as surface integrity are regarded as the indicators with paramount importance. The higher productivity using conventional machining methods demands higher cutting speeds. However, in the case of most of the titanium-based alloys, higher speeds are not feasible due to the greater tool wear caused as a result of the abnormally quick chipping and plastic deformation of the cutting edge. This is primarily due to the properties such as higher strain hardening, toughness as well as pseudo-elastic behavior [6]. Under such conditions, the conventional cutting becomes highly uneconomical as a result of the high tooling cost. Most advanced tool materials such as coated cemented carbides, cubic boron nitride (CBN), poly-crystalline boron nitride (PCBN), etc., also fall prey to titanium’s ability to reach with almost every material which results in a higher rate of tool failure [7,8]. Moreover, the surface integrity of the conventionally machined titanium parts is also largely affected. The major concerns related to the surface integrity are visible in the form of topology characteristics such as surface roughness (SR) and waviness. Furthermore, the mechanical properties as well as the metallurgical state of the component are also impacted. Such impacts results in additional usage of post-machining processes which further increases the manufacturing cost [9,10]. These concerns and difficulties create a void that is usually filled by certain non-conventional cutting methods for machining titanium and its alloys.

### 1.1. Electric Discharge Machining (EDM) of Titanium and Its Alloys

EDM is a commonly used non-conventional technique for cutting difficult-to-cut materials. Its advantages lie in the fact that it is highly accurate, can cut highly complex shapes and gives a much better surface finish when compared to the conventional alternatives [11]. Additionally, the residual stresses are also lower in the machined surface due to zero contact between the cutting tool and the work piece [12]. WEDM is one of the most commonly used EDM techniques due to the numerous options in the types of wires to be used. Additionally, it also fulfils the sustainability approach with the use of eco-friendly di-electric fluids such as distilled water. Furthermore, the continuous travel of the wire does not affect the machined surface in an uneven manner, further improving the dimensional accuracy and machining performance. A highest material removal rate (MRR) equal to 33.6 mm^3^/min can be achieved in this process [13]. However, the surface finish is much higher at lower MRR. The cutting speed (CS) surge also linearly decreases the surface finish with drastic deterioration beyond the speed of 2.65 mm/min.

Nourbakhsh et al. (2013) performed research on WEDM of Ti6Al4V alloy using two different wire electrodes: high-speed brass and zinc-coated brass. The Taguchi’s L_18_ array was adopted to vary input parameters. It was concluded that WT, WF, injection pressure and voltage had no effect on the CS. However, the surge in the peak current and pulse interval did increase the CS [14]. Alias et al. (2012) studied the effect of three different WFs (6 mm/min, 4 mm/min and 2 mm/min) along with other electrical settings on the output parameters. It was found that at WF of 4 mm/min, wire speed of 8 m/min, WT of 1.4 kg and SV of 60 V gave the best results in terms of surface finish. Further, the importance of electrical settings was stressed due to their ability to impact the surface finish as a result of the arcing phenomenon [15]. Sivaprakasam et al. (2014) also studied the influence of WEDM parameters on responses such as surface roughness (SR), kerf width and MRR while machining Ti-6Al-4V alloy. It was concluded that the most optimal setting of input parameters was 100 V (voltage), 10 Nf (capacitance) and 15 µm/s (feed rate) [16]. Majumdar and Maity (2019) in a recent study conducted process capability index (CP_i_) calculations for output parameters such as surface roughness and MRR while machining titanium grade 6 alloy. The WEDM input parameters assumed were A_ON_, A_OFF_, WF and WT. With the aim of minimizing the process capability index, the optimum parametric settings for MRR were found out as: A_ON_ = 115 µs, A_OFF_ = 55 µs, WT = 6 kg f and WF = 4 m/min, while for SR, the optimum settings were: A_ON_ = 105 µs, A_OFF_ = 60 µs, WT = 5 kg f and WF = 4 m/min [17].

Therefore, it may be concluded that the input parameters such as A_ON_, A_OFF_, peak current, etc., have a direct impact on the SR values while machining titanium alloys. Similarly, the parameters such as peak voltage, water pressure and wire feed (WF) have negligible influence on SR. Moreover, with a view of improving the efficiency of the experimental investigations, there is a need of effective predictive and optimization techniques in order to minimize the number of trials. The following section shall discuss the literature on such optimization techniques while conducting WEDM studies.

### 1.2. Optimization Techniques for WEDM Process Parameters

A majority of the research in the area of WEDM has been concentrated towards the optimization of the numerous process parameters discussed in the previous sections. Techniques such as Response Surface Methodology (RSM), Grey’s Relational Analysis (GRA), Genetic Algorithms (GA), Artificial Neural Networks (ANN), Taguchi’s methods etc., have been extensively used for the optimization of process parameters. Prasanna et al. (2019) used GRA for multi-objective optimization (MOO) of MRR and SR while machining titanium 6242 alloy. The input parameters such as A_ON_, A_OFF_, SV and WF were varied. It was concluded that the higher SV and WF led to increased SR values, which was further confirmed by Scanning Electron Microscopy (SEM) imaging [18]. Chaudhary et al. (2019) also used an integrated RSM-GRA approach for the MOO of WEDM parameters while machining pure titanium. The priorities were set in maximizing the cutting rate and minimizing the SR while varying parameters such as A_ON_, A_OFF_ and discharge current. Ultimately, A_ON_ = 6 µs, A_OFF_ = 4 µs and discharge current of 6 A were found out as the optimal parametric combination [19]. Thangaraj et al. (2020) in a recent study used the Taguchi-GRA (TGRA) combinational approach for optimizing the surface quality while machining titanium (α-β) alloy. The surface quality was expressed as a combination of multiple aspects such as recast layer thickness, micro-cracks, sub-surface formation and microhardness. It was shown that SV of 70 V, discharge current (Ip) of 15 A and duty factor of 0.6 gave the best results. Furthermore, the wire electrode was concluded as the dominant parameter in terms of quality evaluation [20].

Many researchers have directly used RSM and desirability-based approaches for MOO of WEDM process parameters [21,22,23,24]. Priyadarshini et al. (2019) made use of the TOPSIS approach for parametric optimization while machining Ti6Al4V alloy. The Ip was the most significant parameter in affecting MRR, tool wear rate and surface quality [25]. In pursuit of developing effective predictive models, Maity and Majumdar (2018) compared the general regression neural network (GRNN) and multiple regression analysis (MRA) models for their effectiveness in predicting the key parameters such as SR, kerf width and MRR. It was found out that the predicted response for GRNN had a maximum 5% error while the MRA had nearly 10% error values. Therefore, the GRNN was concluded as a more reliable model for predicting the output parameters while machining titanium grade 6 alloy [26]. The same authors in another experimental study used the combinational approach of MOO on the basis of Ratio Analysis (MOORA) and Principal Component Analysis (PCA) for optimizing the process parameters such as the average SR, average kerf width and average cutting speed. The proposed combinational approach yielded more accurate and improved results [27]. Regarding the processing of Ti-alloys, Table 1 describes the recent research, input and output parameters, methodology adopted and findings.

There is a lot of research conducted on titanium alloy using different planning of experiments and optimization techniques. The optimization can be done by statistical techniques or by artificial techniques. The work done by Gupta et al. [28] used the RSM technique for planning as well as for optimization. The benefit of this technique is the development of empirical models, but no technique is used to solve the empirical model. In the research conducted by Goyal et al. [29], ANN and NSGA-II were used for the optimization purpose. In this research, the main drawback is the lack of planning of experiments; however, modelling and optimization are present. Similarly, in every research conducted on titanium alloys, the steps involved are planning of experiments, analysis, modelling and optimization. In every research, demonstrated in Table 1, either one or two points are missing. Therefore, to fill this gap, the EDAS-PSO method was proposed. In the present work, the planning of experiments was done using a Taguchi-based L16 orthogonal array. After that, all the output parameters were converted into a single response known as the performance index. The ranking of the experimental setting was completed using the EDAS method. This step was adopted to select the best setting out of all the available settings, which is essential in many cases when there is involvement of many responses. In the next step, the modeling of the performance index is completed using regression analysis. The developed model is further solved by PSO to get the optimized setting. Therefore, in the proposed method, the steps involved are planning of experiments, analysis, normalization, evaluation of the performance index, ranking, empirical model development and optimization.

The above-mentioned studies portray the extensive research being pursued across the world by using several optimization techniques for boosting the efficacy of the WEDM process. Conclusively, it would be fair to state that surface roughness is mostly influenced by T_ON_, T_OFF_, servo voltage and discharge current. Moreover, numerous research studies are available with techniques such as RSM, Desirability, GRA, GA, etc. However, limited work has been published on the machining of pure titanium (Grade 2). The parametric optimization of WEDM using a combination of statistical and artificial techniques is another criterion of research. The current study shall therefore focus on hybrid optimization techniques such as EDAS-PSO.

## 2. Experimental Procedure and Data Collection

In the present work, pure titanium (Grade 2) was machined at different combinations of process parameters of WEDM such as A_ON_, A_OFF_, SV and WT, while dimensional accuracy (DA), Average SR (R_a_) and Root mean square SR (R_z_) were taken as the output parameters. The Table 2 and Table 3 enlist the composition of titanium (Grade 2) and the input parameters with their levels.

**Figure 1:**
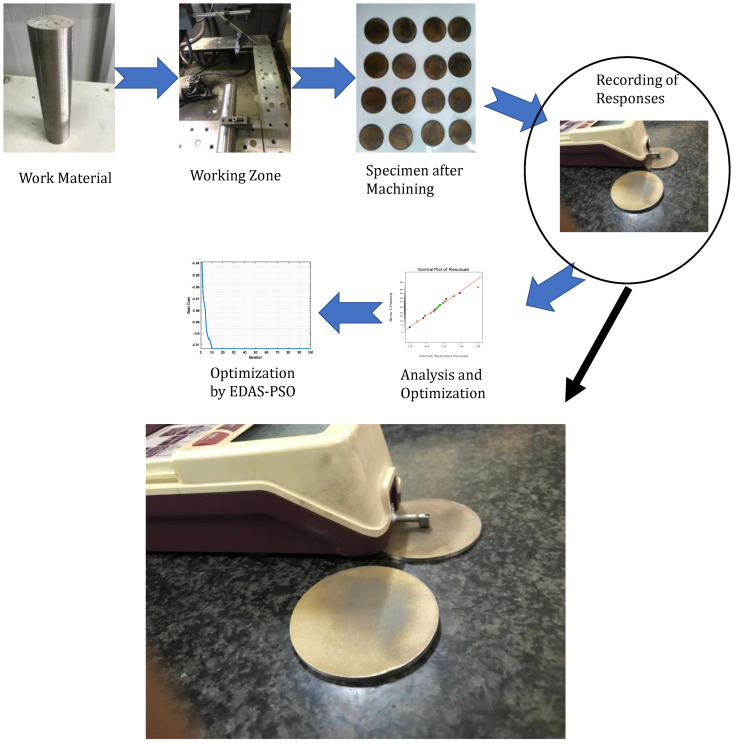
Process flow in the present research.

The experiments were designed for four parameters using a two-level design. Therefore, a total of 16 experiments were designed using a two-level factorial design and the corresponding values of response characteristics were evaluated. Each experiment is repeated twice and the average value of the experiments is indicated in Table 3. To maintain statistical accuracy. The range of input parameters was selected by performing preliminary pilot experimentation as well as on the basis of the extensively researched literature. The experimentation was conducted on Excetek WEDM. Four significant input parameters viz.: A_ON_, A_OFF_, SV and WT were considered for machining the titanium bar. The zinc-coated brass wire with 0.25 mm diameter was used as the electrode for the cutting purpose. Deionized water was used to flush away the debris particle generated during the machining process. A pure titanium cylindrical bar of 25 mm diameter with 300 mm length was used for the experimentation. Dimensional accuracy (DA), mean roughness depth and average SR were chosen as the response characteristics evaluated in current research work. The dimensional accuracy was measured by a Mitutoyo make micrometer with least count of 0.001 mm. Three readings were taken at different locations on a flat disk (as shown in Figure 1). The SR of the disk was measured at three different locations after cleaning it with acetone. The SR was measured using a Mitutoyo make SR tester (model: SJ-201P). Another response evaluated in the present work is the weight loss of wire (wire loss) and reduction in wire diameter (DR). These two responses are related to the wire characteristics. In the first characteristic, i.e., wire loss, 60 mm length of unused wire and the same length of used wire after each experiment were weighted. Three samples of used wire were chosen and the average of the differences of three values were selected for the analysis purpose. In the second characteristic, reduction in the wire diameter values was computed using the Mitutoyo make micrometer. Initially, the fresh wire diameter was measured at three different places and the average of these three values was recorded. After that, used wire was selected after each experiment. The diameter of used wire was measured at three different places. The average of these three values (used wire) is subtracted from the average of three values (fresh wire) and the difference of this is used in the current research.

## 3. Methodology Adopted

**Figure 2:**
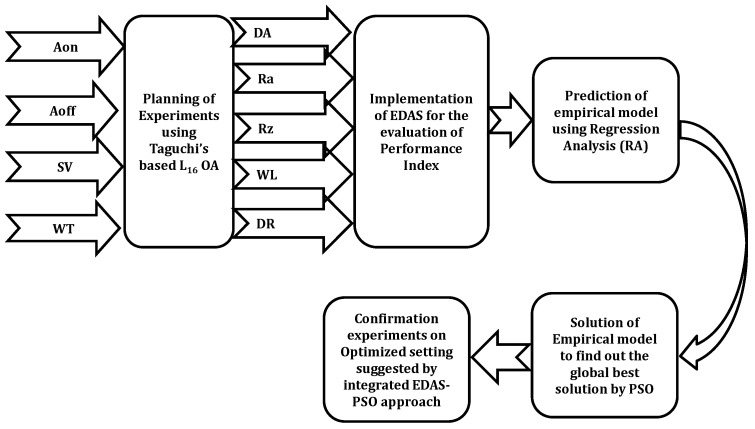
Methodology adopted in the present research work.

In the present research, an integrated approach of EDAS-PSO is used to solve the present MCDM problem (Figure 2).

Here, the planning of experiments was done using a Taguchi-based L16 orthogonal array. Then, the normalization was done using the EDAS method and all the responses were converted into a single response known as the performance index. The regression analysis was performed to develop the empirical model of the performance index with respect to the input parameters. After that, the empirical model is solved with the help of PSO.

### 3.1. Evaluation Based on Distance from Average Solution (EDAS)

Keshavarz Ghorabaee et al., initially developed EDAS, which is used to solve the MCDM problems of inventory management. Most of the inventory problems are based on a single criterion, but to solve the multi-criterion problems EDAS was developed, which provides the consistent solutions. Different researchers in uncertain conditions already used some extensions of EDAS. In MCDM problems, the appropriate weights are provided to each response variable depending upon their importance. If equal importance is given to each response, then equal weight is provided to each response. However, the sum of all the weights is equal to one. In the EDAS method, the negative and positive distances from average solution is evaluated. The steps involved in the investigations of EDAS are given below:

Step 1: The decision matrix is developed

(1) $$P={\left[P_{ij}\right]}_{m\times n}=\left[\begin{array}{cccc} P_{11} & P_{12} & . & P_{1n}\\ P_{21} & P_{22} & . & P_{2n}\\ . & . & . & .\\ P_{m1} & P_{m2} & . & P_{mn} \end{array}\right]\;$$

Here *P_ij_* is the performance value of the *i*th alternative corresponding to the *j*th criterion, n is the number of attributes and m is the total alternative numbers.

Step 2: To find out the average solution corresponding to all solutions

(2) $$\bar{P}={\left[\bar{P_{j}}\right]}_{1\times n}\;\left(j=1,2,\ldots \ldots \ldots ,n\right)$$

where

$$\bar{P_{j}}=\frac{\sum_{i=1}^{m}P_{ij}}{m}\;\left(j=1,2,\ldots \ldots \ldots .,n\right)$$

Step 3: In the next step, the negative distance from average (*NDA*) and positive distance from the average (*PDA*) are evaluated depending upon the type of attribute.

(3) $$PDA={\left[PDA_{ij}\right]}_{m\times n}\;NDA={\left[NDA_{ij}\right]}_{m\times n}$$

If the *j*th response variable is higher-the-better type

(4) $$PDA_{ij}=\frac{\max\left(0,\;\left(p_{ij}-\bar{P_{j}}\right)\right)}{\bar{P_{j}}}\;$$

(5) $$NDA_{ij}=\frac{\max\left(0,\;\left(\bar{P_{j}}-p_{ij}\right)\right)}{\bar{P_{j}}}\;$$

If *j*th response is lower-the-better type quality attribute

(6) $$PDA_{ij}=\frac{\max\left(0,\;\left(\bar{P_{j}}-p_{ij}\right)\right)}{\bar{P_{j}}}$$

(7) $$NDA_{ij}=\frac{\max\left(0,\;\left(p_{ij}-\bar{P_{j}}\right)\right)}{\bar{P_{j}}}\;$$

Step 4: In step 4, the weighted sum of NDA and PDA is calculated:

(8) $$SP_{i}=\frac{1}{m}\overset{n}{\underset{j=1}{\sum }}w_{j}PDA_{ij}\;$$

(9) $$SN_{i}=\frac{1}{m}\overset{n}{\underset{j=1}{\sum }}w_{j}NDA_{ij}\;$$

Step 5: In the next step, the normalization of the SP and SN is carried out using Equations (10) and (11):

(10) $$NSP_{i}=\frac{SP_{i}}{\max_{i}\;\left(SP_{i}\right)}$$

(11) $$NSN_{i}=1-\frac{SN_{i}}{\max_{i}\;\left(SN_{i}\right)}\;$$

Step 6: In this step, the average of all the alternatives is computed as the Appraisal Score (AS):

(12) $$AS_{i}=\frac{1}{2}\left(NSP_{i}+NSN_{i}\right)$$

where the value of *AS_i_* should be in between 0 and 1. A higher value of AS shows the preference for one alternative over the other.

### 3.2. PSO

Particle swarm optimization (PSO) is inspired by bird flocking or the social behavior of bee swarming and was developed by James Kennedy and Russell C Eberhart [36]. In this methodology, a set of particles are flown in n-dimensional search space, which are randomly distributed. These particles learn from their previous experiences. All the co-ordinate values are compared with each other and the best coordinate particles are known as global best values (gbest). In these, two best values exist: one is the individual best of particles known as particle best (pbest), while the other is global best (gbest). The two factors are velocity and position of particles and these factors are upgraded with each iteration. It is also known for fast speed, high dependability and good robustness, which is beneficial to search the local optima in case of multi-response optimization. The main steps involved in the implementation of PSO are as follows:

1. Initialize the population by creating random permutations;
2. Using the weights, the score of each permutation is evaluated;
3. Non-dominated permutations are identified, and archives are updated accordingly;
4. In the next step, updating of gbest and pbest take place;
5. For each particle, the leader permutation is selected as per the technique;
6. The upgraded values are noted for position and velocity of particles and the best values are selected as the solution. In the search space, the velocity and position of the ith particle are shown as *w_i_* = (w_i1_, w_i2_, …, w_in_) and x_i_ = (x_i1_, x_i2_, …, x_in_), respectively. The values of position and velocity are upgraded using Equations (13) and (14) [36]:

(13) $$w_{i}^{k+1}=vw_{1}^{k}+c_{1}r_{1}\left(p_{i}-x_{i}^{k}\right)+c_{2}r_{2}\left(p_{g}-x_{i}^{k}\right)$$

(14) $$x_{i}^{k+1}=x_{i}^{k}+w_{i}^{k}\;$$

In these equations, $w_{i}^{k+1}$ and $x_{i}^{k+1}$ are the updated velocity and position of the ith particles, v is the inertia weight, r_1_ and r_2_ are the uniformly distributed random numbers, c_1_ and c_2_ are the cognitive and social parameters (positive parameters), p_i_ is individual best and g_best_ is global best;

7. Move the particle according to the Equation (14); if the condition is not satisfied, then the algorithm is repeated from step 2.

In the present research, the optimization has been set up by balancing in between the accuracy, Ra, Rz, WL and DR. It is very complex for the manufacturing engineers to integrate EDAS-PSO and optimize the responses for WEDM. Due to the following reasons, the EDAS coupled with PSO is proposed in the present research. First, the optimization of more than two responses of WEDM while machining pure Ti is not processed by any metaheuristic approach. Second, the Taguchi method is an effective method for the planning of experiments and exploring the relationship between the input parameters and response variables. The EDAS method is used for the normalization and calculation of appraisal score (AS). Then, engineers for various engineering applications solve the empirical relation of input parameters with the performance index using the PSO algorithm. Third, there is limited study on the Taguchi method coupled with EDAS-PSO by researchers on WEDM. The EDAS technique individually provides the ranking of the experimental setting. However, it becomes necessary to optimize the parameters with the given range for output parameters. Therefore, it is valuable to use an integrated approach for the optimization of process parameters.

## 4. Results and Discussion

The results corresponding to planned experiments are shown in Table 4. The ANOVA [37] for the different responses is evaluated and the results are discussed in this section.

### 4.1. Analysis for Response Characteristics

Table 5 gives the statistical summary for dimensional accuracy; it is observed that A_on_ has the maximum influence of 44.67% on dimensional accuracy, followed by interaction of A_on_ and WT (16.11%), A_off_ (5.86%), SV (0.64%) and WT (0.64%).

The sum of squares provides the percentage contribution of each process parameter for evaluating the response variable and the degree of freedom is equal to one value less than the value of level. The value of mean square is obtained by dividing the SS to df. The larger the F-value in the ANOVA table, the larger is the contribution of process parameter in the process. The *p*-value also defines the significance of the parameter. For the least value of *p* of a parameter in the ANOVA table, the contribution of that particular parameter is maximum in the process [37].

**Figure 3:**
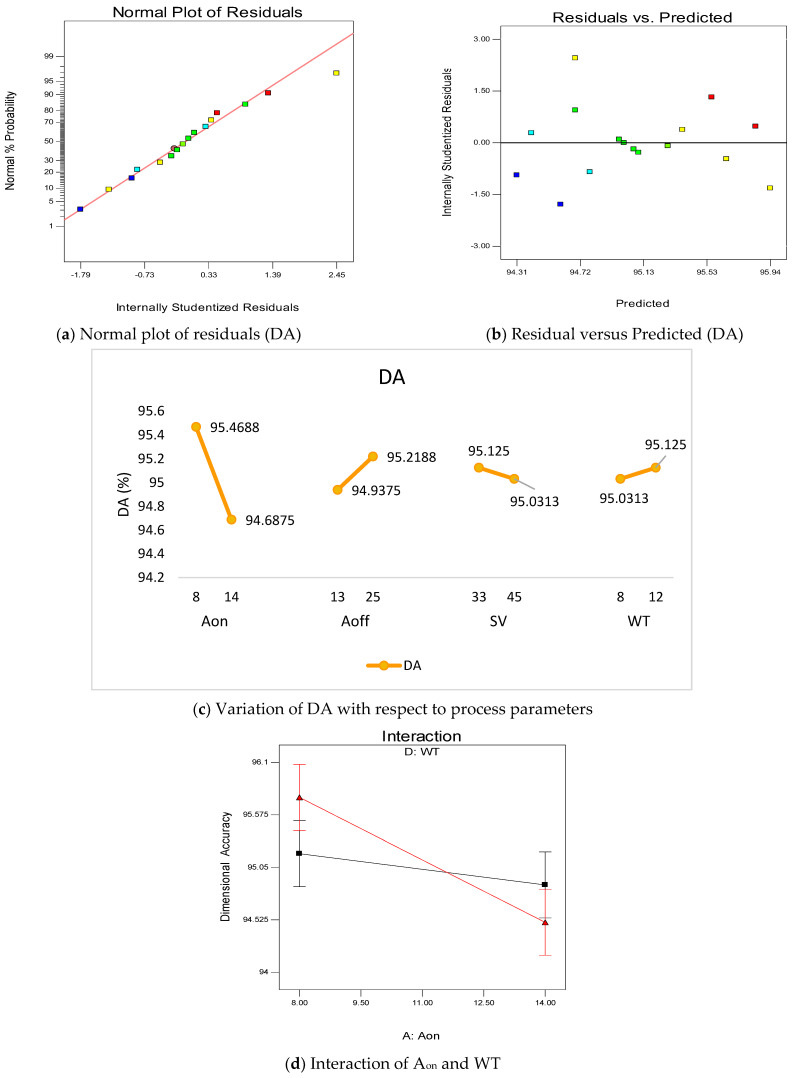
Summary of process parameters with respect to dimensional accuracy.

Figure 3 demonstrates the overall summary of DA with respect to the input parameters viz.: A_on_, A_off_, SV and WT. Figure 3a represents the normal plot of residuals, which signifies that all the residuals are on a straight line. By the help of this test, normality is verified. Figure 3b shows the residuals versus the predicted curve. For a good model, these residuals are randomly distributed. In the present model of DA, all the residuals are randomly distributed, which shows the presence of a good model. Figure 3c illustrates the variation of DA with respect to A_on_ and it was found that with the increase in A_on_ value, the DA decreases. The main reason for this is the higher current on-time in the circuit, due to which the discharge power increases. This high discharge power increases the crater size and decreases the DA. Thus, a low value of A_on_ favors the DA due to precise removal of craters from the surface [35]. A high value of A_off_ favors the DA; as the A_off_ increases in the circuit, the current off-time in the circuit also decreases, which decreases the discharge power. The low amount of discharge power decreases the crater size from the surface. Therefore, material is removed from the surface more precisely, which improves the DA [38].

Figure 3c provides the variation of DA with respect to SV. It is clear from Figure 3c that the DA decreases with the increase in SV value. The probable reason for reducing the accuracy with increases in SV is the increment in the voltage at the inter-electrode gap (IEG). However, it has been found that the DA value increases with the increase in WT value. This is due to the removal of deflections from the wire with the increase in WT. These deflections may cause the non-uniform spark generation by which the DA decreases. Therefore, with the increase in WT, the deflection is reduced and DA increases [39,40]. Figure 3d shows the interaction plot between A_on_ and WT. It is clear from Figure 3d that there is a significant interaction between two parameters as both lines strongly intersect each other. Equation (15) shows the empirical relation between dimensional accuracy and input parameters of WEDM.

Dimensional Accuracy = +91.83854 + 0.26042 × A_on_ + 0.023437 × A_off_ − 0.00781250 × SV + 0.45312 × WT − 0.039062 × A_on_ × WT (15)

### 4.2. Mean Surface Roughness

Table 6 shows the ANOVA for Ra and the statistical summary of Ra for the machining of titanium by WEDM. It is clear from the analysis that A_on_ plays a pivotal role for the investigation of Ra with a percentage contribution of 94.11%. The percentage contribution of all other parameters (such as A_off_, SV and WT) is negligible as compared to A_on_. However, the model is significant for the investigation of Ra. The statistical terms such as MS, F-value and *p*-value support the analysis.

**Figure 4:**
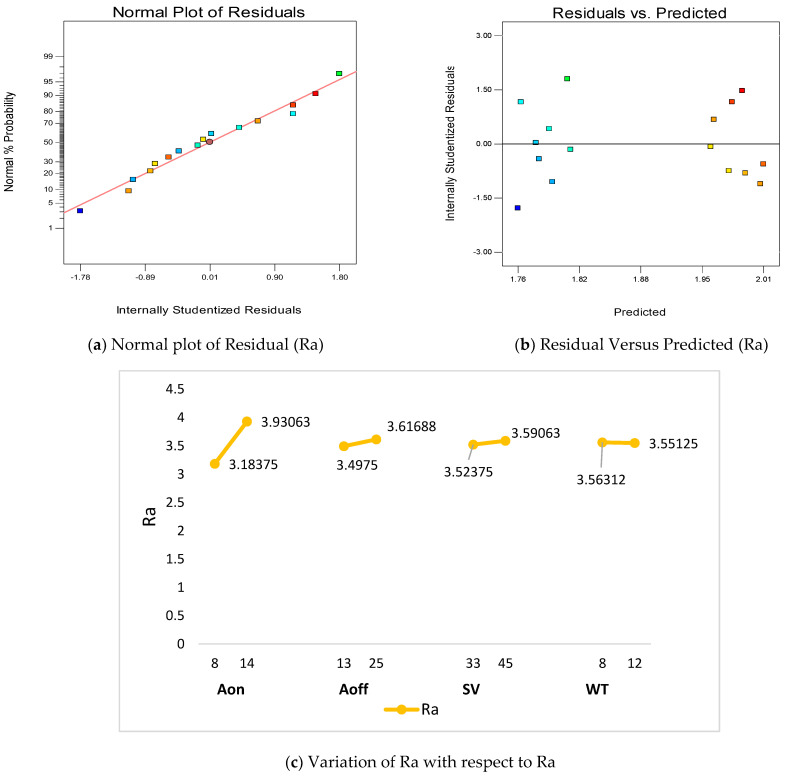
Summary of process parameters with respect to Ra.

Figure 4a illustrates the normality plot of residuals for mean surface roughness. All the residuals are observed on a straight line and verifies the normality test. Another ANOVA test is the residual versus predicted test (Figure 4b), which represents a good ANOVA model. In this test, all the residuals are randomly distributed, which shows a good model. Figure 4c presents the variation of Ra with A_on_ and it is found that the value of Ra increases with increase in the A_on_ value. This is due to high discharge power, which increases the crater size and increases the Ra value [40]. It is observed from Figure 4c that amplification in A_off_ values increases Ra values. This is due to high A_off_ values in the circuit decreasing the discharge power; consequently, crater sizes on the surface decrease and hence Ra values. The increase in SV increases the Ra value. This spark voltage in the IEG increase the discharge power and this ameliorates the Ra value. Thus, a low value of SV favors the surface quality [30]. A high value of WT also increases the surface quality by eliminating the deflections from the wire electrodes. Equation (16) shows the empirical relation between Ra and input parameters of WEDM.

Final equation in terms of actual factors:

Ra = +1.81125 + 0.12448 × A_on_ + 9.94792 × 10^−3^ × A_off_ + 5.57292 × 10^−3^ × SV − 2.96875 × 10^−3^ × WT (16)

### 4.3. Mean Roughness Depth

Table 7 gives the ANOVA for Rz and it is found that the *p*-value of the model is less than 0.05, which confirms the significance of the empirical model. A_on_ has the maximum contribution on Rz with 89.9% contribution. Process parameters such as A_off_, SV and WT have *p*-values greater than 0.05; therefore, these parameters play insignificant roles for the investigation of Rz. From the analysis of tabulated F-values, the maximum influence on Rz is of A_on_, followed by SV, A_off_ and WT.

**Figure 5:**
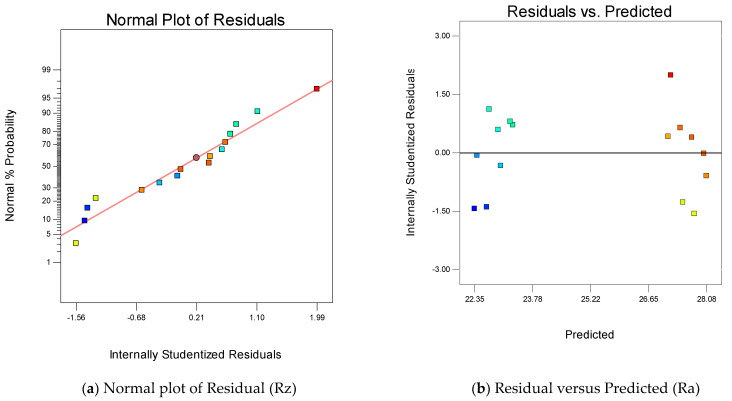

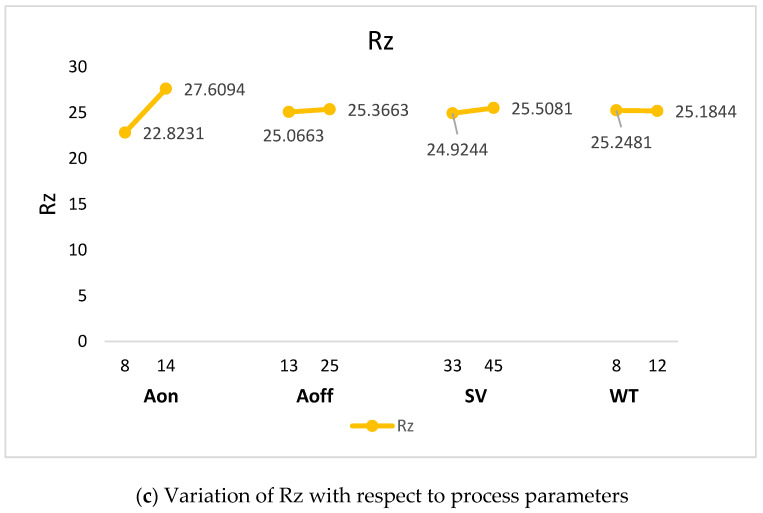
Summary of process parameters with respect to Rz.

Figure 5a,b represents the normality test and residual versus predicted test for the verification of a good ANOVA. The residuals position in both plots are as per the significance of good ANOVA. The variations of Rz with A_on_, A_off_, SV and WT are provided in Figure 5c. It is clear from Figure 5c that the value of Rz increases with increase in the A_on_ value. This is due to high discharge power, which increases the crater size and increases the Rz values [40]. It is observed from Figure 5c that amplification in A_off_ values increases the Rz value. This is due to the fact that high A_off_ values in the circuit diminish the discharge power; then the crater sizes on the surface decrease and hence Rz values. The increase in SV increases the Rz value, which is due to increase in the IEG by increasing the SV value and enhancing the Rz value. Thus, a low value of SV favors the surface quality [30]. A high value of WT also increases the surface quality by eliminating the deflections from the wire electrodes. Equation (17) gives the empirical relation between Rz and input parameters of WEDM.

Rz = +14.22865 + 0.79771 × A_on_ + 0.025000 × A_off_ + 0.048646 × SV − 0.015937 × WT (17)

### 4.4. Wire Loss (WL) and Reduction in Wire Diameter (DR)

**Figure 6:**
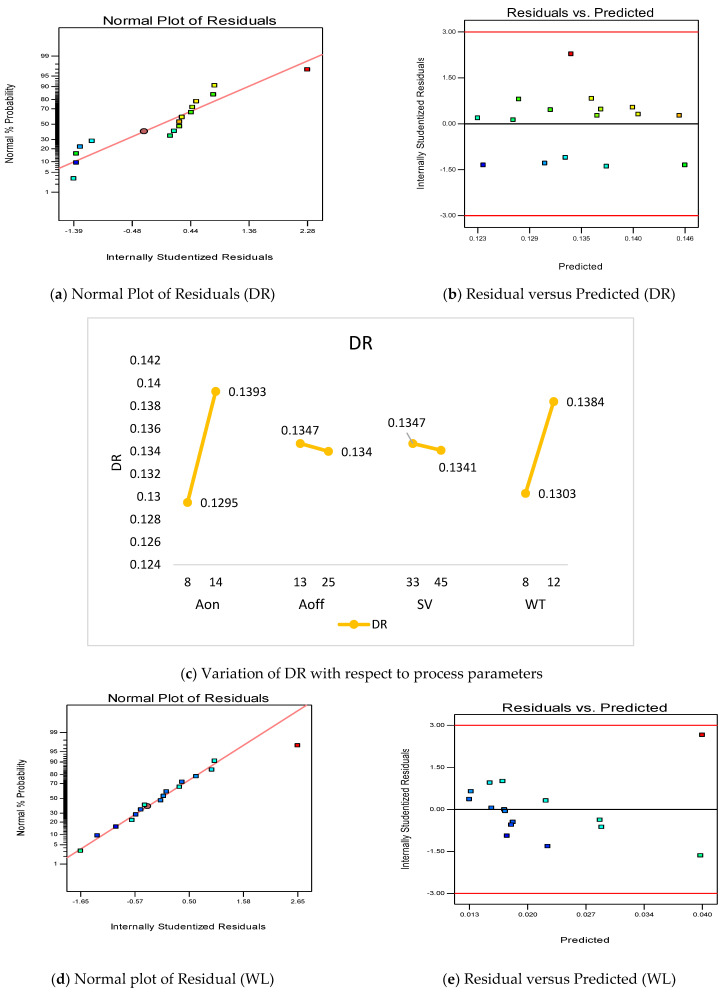

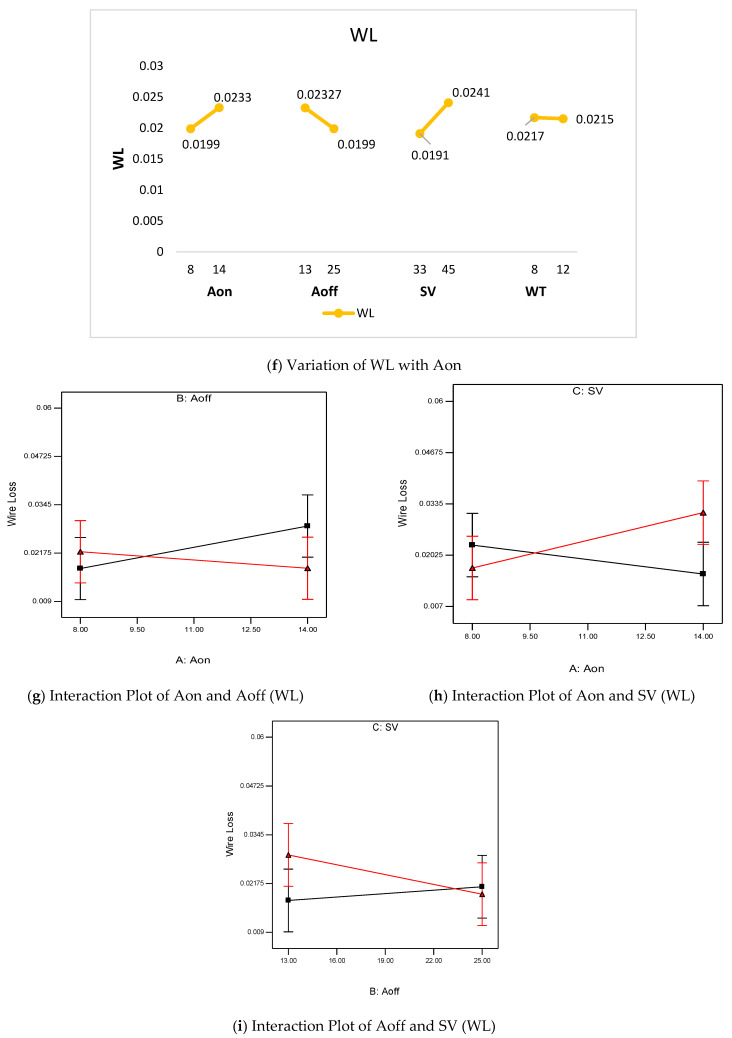
Summary of process parameters with respect to WL and DR.

The analysis of WL and DR is presented in Figure 6. Figure 6a shows the normal distribution plot of WL and it is observed that all the residuals fall on the straight line, which shows a good ANOVA. Another plot for the verification of ANOVA is the residual versus predicted plot, and it is evident from Figure 6b that all the residuals are randomly distributed, which is required for a good ANOVA. The measurement of the reduction in wire diameter (DR) is already discussed in Section 2. The variation of the DR with respect to the process parameters is depicted in Figure 6c. The value of DR increases (0.1295 mm to 0.1393 mm) with the increase in A_on_ value from 8 µs to 14 µs. The main fact is that at a high value of A_on_, discharge energy is high due to which spark intensity is high [40]. This intensity enhances the DR value upto 0.1393 mm. The value of DR decreases with the increase in A_off_ and SV value. The increase in A_off_ value decreases the current intensity in the circuit, which decreases the discharge power, due to which the removal of craters from the wire electrode also decreases. Thus, the reduction in DR value from 0.1347 to 0.134 is observed. The increase in SV value increases the spark-waiting time in the circuit, whch decreases the discharge power and consequently the reduction in DR value is observed [39]. The value of DR increases from 0.1303 mm to 0.1384 mm with the increase in WT value from 8 N to 12 N. With the increase in WT, the irregularities in the wire are eliminated, due to which the sparks will be uniformly distributed, hence the DR value increases.

Figure 6d exhibit the normal distribution plot of WL (in g), which describes that the residuals are normally distributed. Figure 6e shows the residual versus predicted plot, in which all the residuals are randomly distributed. This verifies the good ANOVA in the present research work. The variation of the WL with respect to the process parameters is presented in Figure 6f. It is clear from Figure 6f that the value of WL increases with the increase in A_on_ and SV values, while WL decreases with the increase in A_off_. The WT has negligible influence on WL as evident from Figure 6f. It has also been observed that there is interaction between A_on_ and A_off_ (Figure 6g), A_on_ and SV (Figure 6h) and A_off_ and SV (Figure 6i). Table 8 gives the ANOVA for DR and WL.

## 5. EDAS-PSO

The procedure for the implementation of EDAS was already described in Section 3. In the first step, the decision matrix is developed as per the Equation (1). After that, average solutions are calculated along with the PDA and NDA according to Equation (4) to Equation (7) (Table 9), depending upon the attribute of response variables. In the next step, the weights are given to each response variable according to their importance. However, the sum of all the weights should be equal to 1. In the present research, the number of response variables is five; thus, to provide equal importance to each response variable the value of each weight is 0.2. To give more importance to any response, more weight is provided to the specified response. For example, one weight is 0.4 and the other weights (four responses) are 0.15 each. In the next step, the weights are multiplied by PDA and NDA to find out the weighted NDA and PDA (Table 10). The normalization is done using Equations (10) and (11) and shown in Table 11. The appraisal score (AS) is calculated using Equation (12) and is also given in Table 11. The maximum value of AS is given a rank of 1 and the minimum value is given a rank of 16. The process parameter setting corresponding to the maximum AS value provides the optimized setting for all the response variables.

In the next step, the AS calculated by the EDAS method is considered as a response and is modelled according to the regression analysis considering A_on_, A_off_, SV and WT as input parameters. The empirical model is developed and regression coefficients are calculated. The empirical model is shown in Equation (18):

AS = +1.44288 − 0.037201 × A_on_ − 4.23625 × 10^−3^ × A_off_ + 3.78521 × 10^−3^ × SV − 0.043355 × WT (18)

The developed empirical model is solved using PSO. Initially, the objective function is developed as an ‘m’ file in matlab by the mathworks language. In the PSO, two best solutions were predicted: one is individual best and another is global best. The global best solution is the solution at which the maximum value of the response is predicted and the position is the setting of the input parameters [41]. As the response is ‘AS’, which is the larger-the-better type. Thus, in PSO to optimize AS, the objective function is

$$AS=-\left(Obj.\;function\right)$$

The limits of the process parameters to solve the empirical models are given by Equation (19) to Equation (22):

8 ≤ A_on_ ≤ 14 (19)

13 ≤ A_off_ ≤ 25 (20)

33 ≤ SV ≤ 45(21)

8 ≤ WT ≤ 12(22)

The computation was processed using an Intel i5 processor with 8 GB ram. The average processing time to get the solution was 23 s (average of five trials). The parameters to solve the PSO are: maximum and minimum values of inertia, 0.4 and 0.1, respectively; acceleration coefficients C1 and C2 are equal to 1; number of iterations, 100; swarm size, 50.

**Figure 7:**
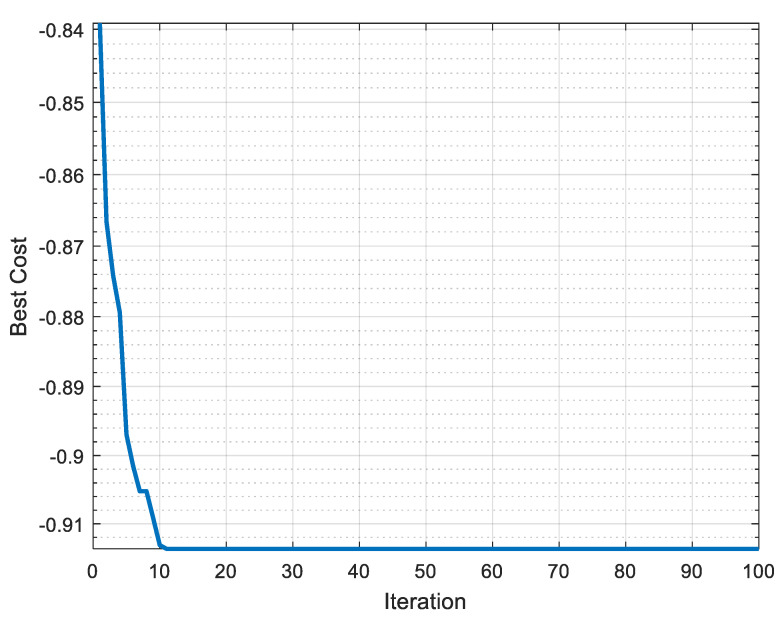
Pareto-optimal front after PSO.

Figure 7 depicts the best solution investigated with the number of iterations. It is clear from the Figure 6 that the best AS value is 0.9137, which is obtained after 11 iterations. After 11 iterations, the value of AS remains constant, thus a straight line is observed. The optimized setting predicted after EDAS-PSO is: A_on_: 8 µs; A_off_: 13 µs; SV: 45 V; and WT: 8 N. The values of DA, Ra, Rz, WL and DR are predicted and then the validation experiments are performed at the optimized setting suggested by EDAS-PSO (Table 12). It was observed that in the case of responses, the predicted values are in line with the experimental values. The response variables obtained at the optimized setting are compared with the trial run number 3, i.e., A_on_: 8 µs; A_off_: 25 µs; SV: 33 V; and WT: 8 N. The value of AS corresponding to trial run number 3 is 1. The response variables with the trial run number 3 are comparable with the response variable investigated at the optimized setting suggested by EDAS-PSO.

It was observed that the experimental values have a close agreement with predicted values at the suggested solutions. Thus, the proposed technique can be successfully implemented for the optimization of the process parameters of WEDM while machining titanium alloys.

## 6. Morphological Investigations

**Figure 8:**
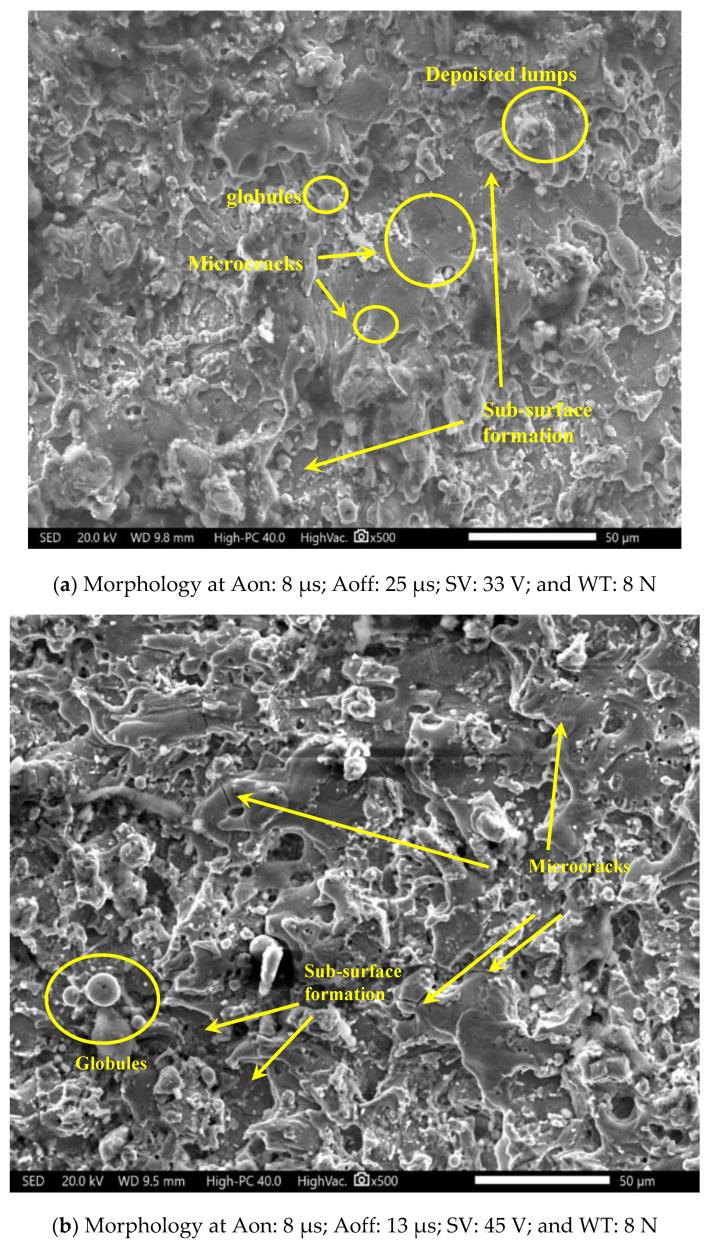
Morphology of machined surface.

Figure 8 presents the morphological investigations of the machined surfaces at different machining conditions.

A high value of A_on_ and low value of A_off_ are considered as high energy parameters, while a low value of A_on_ and high value of A_off_ are assumed as low energy parameters. Therefore, Figure 8a is the morphology of the surface obtained at low discharge parameters. It is evident from Figure 6a that initially material was melted and then the rapid cooling due to the dielectric became the reason for microcracks. In this case, the thermal stresses become greater than the fracture strength and crack formation takes place [42]. The lumps are observed on the surface, due to the deposition of the melted materials. The main function of the dielectric is to eliminate the debris from the working area, but if the time between two pulses is less then lumps are observed to be deposited on the machined surface [43]. The large amount of discharge energy removes large craters from the machined surface and forms the sub-surface. In Figure 8a, the surface defects are less as compared to the surface defects observed in Figure 8b. The main reason for this is the large discharge energy parameters in Figure 8b. The large amount of discharge creates a large number of defects. These defects include deposited lumps, globules, microcracks and sub-surface formations.

## 7. Conclusions

After the machining of pure titanium by WEDM at different settings of process parameters (A_on_, A_off_, SV and WT), the following conclusions are drawn:

1. The pure titanium is machined successfully using WEDM at different parametric settings.
2. From the ANOVA, it is found that A_on_ is the major influencing factor for the evaluation of DA, Ra, Rz, WL and DR. The DA decreases with the increase in A_on_ value, while Ra, Rz, WL and DR values increases with the increase in A_on_ value.
3. The statistical summary suggests that the models developed for DA, Ra, Rz, WL and DA are significant, while lack of fit are non-significant. These tests verified the presence of a good ANOVA.
4. The multi-response optimization for the optimal solution is predicted using the integrated approach of EDAS-PSO. The optimal setting for the machining of Ti is: A_on_: 8 μs; A_off_: 13 μs; SV: 45 V; and WT: 8 N. The suggested predicted solution at the optimized setting is: DA: 95%; Ra: 3.163 μm; Rz: 22.99 μm; WL: 0.0182 g; and DR: 0.1277 mm.
5. The morphology of the machined surface indicates the presence of deposited lumps, microcracks, sub-surface formation and globules. At the optimized setting suggested by EDAS-PSO, the number of defects on the machined surface is reduced significantly.
6. The SEM micrographs show that the more compact structure was obtained at the optimized setting due to smaller grain formation, which forms the more refined material.

The proposed method of optimization can be implemented for the investigation of other responses viz.: cutting rate, geometrical error, residual stresses, etc. The proposed method of optimization can also be used for the optimization of other manufacturing processes such as ultrasonic machining, laser beam machining, abrasive jet machining, abrasive flow machining, abrasive water jet machining, etc.

## Figures and Tables

**Table 1:** Recent research conducted on titanium alloys.

Sr. No.	Author	Year	Materials	Input Parameters	Output Parameters	Methodology	Finding
1	Gupta et al. [28]	2021	Ti6Al4V	SV, WF, wire tension	CS and surface characterization	RSM	The maximum value of CS 1.75 mm/min
2	Goyal et al. [29]	2021	Ti6Al4V	Ton, Toff, WF, peak current	MRR, wire wear ratio	ANN-NSGA-II	The maximum error between the predicted and actual value is 7.5%.
3	Thangaraj et al. [20]	2020	Titanium (α-β) alloy	gap voltage, duty factor, discharge current	microhardness, WWR, average white layer thickness	TGRA	The optimal settings significantly affect the surface quality.
4	Chaudhari et al. [19]	2020	Pure Titanium	Ton, Toff, discharge current	CR, SR	RSM-GRA	A close agreement between the predicted and actual values has been obtained.
5	Sharma et al. [30]	2021	Ti6Al4V	Ton, Toff, SV	MRR, Rz	Grey-Harmony Search	The optimized value of MRR and Rz at the suggested setting are 6.5 mm^3^/min and 13.84 um.
6	Majumdar and Maity [17]	2020	Titanium Grade 6	Ton, Toff, WF and WT	MRR and SR	Taguchi and Process Capability index	With the proposed approach, the cost of item failure decreases.
7	Farooq et al. [31]	2020	Ti6Al4V	SV, WF, Ton, Toff	Corner radii and geometric deviation	Taguchi	At optimized setting, geometric deviation is minimum
8	Fuse et al. [32]	2021	Ti6Al4V	Ton, Toff, current	CS, MRR and SR	Fuzzy AHP and Fuzzy TOPSIS	The use of fuzzy eliminates the uncertainty from the system.
9	Kumar et al. [33]	2021	Ti Grade 2	Ton, Toff, peak current, SV	White layer thickness, MRR, SR	RSM	The major factors deteriorating the surface are Ton, Toff, SV and peak current.
10	Pramanik et al. [34]	2019	Ti6Al4V	Ton, flushing pressure, WT	MRR, wire degradation, Kerf width, surface generation	Design of Experiments	The recast layer is discontinuous and weak underneath solid layer.
11	Sharma et al. [35]	2019	Ti6Al4V	Ton, Toff, SV	CS, SR	Taguchi Grey relational	The crack intensity increases with the increase in discharge energy.

**Table 2:** Chemical composition of titanium (Grade 2).

Titanium (Grade 2)						
Element	**H**	**N**	**C**	**O**	**Fe**	**Ti**
Content (%)	<0.015	<0.30	<0.08	<0.25	<0.30	>98.9

**Table 3:** Input parameters.

Denotation	Machining Parameter	Level	
		Low	High
A_ON_	Pulse-ON time (µs)	8	14
A_OFF_	Pulse-OFF time (µs)	13	25
SV	Servo Voltage (V)	33	45
WT	Wire Tension (kg F)	8	12
Work piece material		Titanium (Grade 2)	
Work piece dimensions		Cylindrical (25 mm D × 300 mm L)	
Electrode material		Brass (zinc-coated)	
Electrode dimensions		Wire (0.25 mm diameter)	
Electrolyte		Deionized water	

**Table 4:** Design matrix and corresponding results.

Sr No.	A_ON_	A_OFF_	SV	WT	DA (%)	Ra (µm)	Rz (µm)	Weight Loss (g)	DR (mm)
1	8	13	33	8	95	3.205	22.365	0.01505	0.11
2	14	13	33	8	94.5	3.905	28.655	0.01695	0.156667
3	8	25	33	8	95.5	3.115	23.53	0.01085	0.125
4	14	25	33	8	95	3.88	26.57	0.02845	0.121667
5	8	13	45	8	95	3.125	22.755	0.01795	0.135833
6	14	13	45	8	95	4.03	26.64	0.02425	0.123333
7	8	25	45	8	95.25	3.265	23.82	0.01725	0.128333
8	14	25	45	8	95	3.98	27.65	0.01295	0.141667
9	8	13	33	12	95.5	2.925	21.3	0.02415	0.139167
10	14	13	33	12	94.5	3.815	27.44	0.01575	0.1325
11	8	25	33	12	95.5	3.24	21.63	0.02575	0.144167
12	14	25	33	12	95.5	4.105	27.905	0.05925	0.148333
13	8	13	45	12	96	3.155	23.365	0.01415	0.135833
14	14	13	45	12	94	3.82	28.01	0.02435	0.144167
15	8	25	45	12	96	3.44	23.82	0.01615	0.1175
16	14	25	45	12	94	3.91	28.005	0.02235	0.145833
			Average		95.07813	3.55719	25.21625	0.02160	0.13438

**Table 5:** ANOVA for dimensional accuracy.

Source	SS	% Cont.	df	MS	F-Value	*p*-Value
Model	3.71		5	0.74	4.22	0.0253
A-A_on_	2.44	44.67	1	2.44	13.89	0.0039
B-A_off_	0.32	5.86	1	0.32	1.8	0.2094
C-SV	0.035	0.64	1	0.035	0.2	0.6643
D-WT	0.035	0.64	1	0.035	0.2	0.6643
AD	0.88	16.11	1	0.88	5	0.0493
Residual	1.76	32.08	10	0.18		
Cor Total	5.46	100	15			

**Table 6:** ANOVA for Ra.

Source	SS	% Cont.	df	MS	F-Value	*p*-Value
Model	0.16		4	0.041	41.32	<0.0001
A-A_on_	0.16	94.11	1	0.16	159.58	<0.0001
B-A_off_	4.19 × 10^−3^	2.46	1	4.19 × 10^−3^	4.24	0.0639
C-SV	1.40 × 10^−3^	0.82	1	1.40 × 10^−3^	1.41	0.2599
D-WT	4.19 × 10^−5^	0.02	1	4.19 × 10^−5^	0.042	0.8406
Residual	0.011	2.59	11	9.88 × 10^−4^		
Cor Total	0.17	100	15			

**Table 7:** ANOVA for Rz.

Source	SS	% Cont.	df	MS	F-Value	*p*-Value
Model	93.37	-	4	23.34	30.04	<0.0001
A-A_on_	91.63	89.9	1	91.63	117.92	<0.0001
B-A_off_	0.36	0.35	1	0.36	0.46	0.5102
C-SV	1.36	1.33	1	1.36	1.75	0.2122
D-WT	0.016	0.02	1	0.016	0.021	0.8876
Residual	8.55	8.4	11	0.78		
Cor Total	101.92	100	15			

**Table 8:** ANOVA for WL and DR.

**WL**					
**Source**	**SS**	**df**	**MS**	**F-Value**	**Prob > F**
Model	1.10 × 10^−3^	7	1.57 × 10^−4^	1.55	0.275
A-A_on_	4.69 × 10^−5^	1	4.69 × 10^−5^	0.46	0.5153
B-A_off_	4.49 × 10^−5^	1	4.49 × 10^−5^	0.44	0.5243
C-SV	9.90 × 10^−5^	1	9.90 × 10^−5^	0.98	0.3517
D-WT	1.60 × 10^−7^	1	1.60 × 10^−7^	1.58 × 10^−3^	0.9693
AB	2.42 × 10^−4^	1	2.42 × 10^−4^	2.39	0.1609
AC	4.75 × 10^−4^	1	4.75 × 10^−4^	4.69	0.0622
BC	1.92 × 10^−4^	1	1.92 × 10^−4^	1.89	0.206
Residual	8.10 × 10^−4^	8	1.01 × 10^−4^		
Cor Total	1.91 × 10^−3^	15			
**DR**					
**Source**	**SS**	**df**	**MS**	**F-Value**	**Prob > F**
Model	7.35 × 10^−4^	5	1.47 × 10^−4^	0.89	0.5225
A-A_on_	3.84 × 10^−4^	1	3.84 × 10^−4^	2.32	0.1584
B-A_off_	1.56 × 10^−6^	1	1.56 × 10^−6^	9.47 × 10^−3^	0.9244
C-SV	1.56 × 10^−6^	1	1.56 × 10^−6^	9.47 × 10^−3^	0.9244
D-WT	2.64 × 10^−4^	1	2.64 × 10^−4^	1.6	0.2346
CD	8.40 × 10^−5^	1	8.40 × 10^−5^	0.51	0.4919
Residual	1.65 × 10^−3^	10	1.65 × 10^−4^		
Cor Total	2.39 × 10^−3^	15			

**Table 9:** Computation of PDA and NDA.

Sr. No.	PDA					NDA				
1	0.00000	0.00000	0.11307	0.30324	0.18140	0.00082	0.00000	0.00000	0.00000	0.00000
2	0.00000	0.09778	0.00000	0.21528	0.00000	0.00608	0.09778	0.13637	0.00000	0.16589
3	0.00444	0.00000	0.06687	0.49769	0.06977	0.00000	0.00000	0.00000	0.00000	0.00000
4	0.00000	0.09075	0.00000	0.00000	0.09457	0.00082	0.09075	0.05369	0.31713	0.00000
5	0.00000	0.00000	0.09761	0.16898	0.00000	0.00082	0.00000	0.00000	0.00000	0.01085
6	0.00000	0.13292	0.00000	0.00000	0.08217	0.00082	0.13292	0.05646	0.12269	0.00000
7	0.00181	0.00000	0.05537	0.20139	0.04496	0.00000	0.00000	0.00000	0.00000	0.00000
8	0.00000	0.11886	0.00000	0.40046	0.00000	0.00082	0.11886	0.09652	0.00000	0.05427
9	0.00444	0.00000	0.15531	0.00000	0.00000	0.00000	0.00000	0.00000	0.11806	0.03566
10	0.00000	0.07248	0.00000	0.27083	0.01395	0.00608	0.07248	0.08819	0.00000	0.00000
11	0.00444	0.00000	0.14222	0.00000	0.00000	0.00000	0.00000	0.00000	0.19213	0.07287
12	0.00444	0.15400	0.00000	0.00000	0.00000	0.00000	0.15400	0.10663	1.74306	0.10387
13	0.00970	0.00000	0.07341	0.34491	0.00000	0.00000	0.00000	0.00000	0.00000	0.01085
14	0.00000	0.07388	0.00000	0.00000	0.00000	0.01134	0.07388	0.11079	0.12731	0.07287
15	0.00970	0.00000	0.05537	0.25231	0.12558	0.00000	0.00000	0.00000	0.00000	0.00000
16	0.00000	0.09918	0.00000	0.00000	0.00000	0.01134	0.09918	0.11059	0.03472	0.08527

**Table 10:** Computation of Weighted PDA, NDA, SPi and SNi.

Weighted PDA					SPi	Weighted NDA					SNi
0.00000	0.00000	0.02261	0.06065	0.03628	0.11954	0.00016	0.00000	0.00000	0.00000	0.00000	0.00016
0.00000	0.01956	0.00000	0.04306	0.00000	0.06261	0.00122	0.01956	0.02727	0.00000	0.03318	0.08122
0.00089	0.00000	0.01337	0.09954	0.01395	0.12775	0.00000	0.00000	0.00000	0.00000	0.00000	0.00000
0.00000	0.01815	0.00000	0.00000	0.01891	0.03706	0.00016	0.01815	0.01074	0.06343	0.00000	0.09248
0.00000	0.00000	0.01952	0.03380	0.00000	0.05332	0.00016	0.00000	0.00000	0.00000	0.00217	0.00233
0.00000	0.02658	0.00000	0.00000	0.01643	0.04302	0.00016	0.02658	0.01129	0.02454	0.00000	0.06258
0.00036	0.00000	0.01107	0.04028	0.00899	0.06071	0.00000	0.00000	0.00000	0.00000	0.00000	0.00000
0.00000	0.02377	0.00000	0.08009	0.00000	0.10386	0.00016	0.02377	0.01930	0.00000	0.01085	0.05409
0.00089	0.00000	0.03106	0.00000	0.00000	0.03195	0.00000	0.00000	0.00000	0.02361	0.00713	0.03074
0.00000	0.01450	0.00000	0.05417	0.00279	0.07145	0.00122	0.01450	0.01764	0.00000	0.00000	0.03335
0.00089	0.00000	0.02844	0.00000	0.00000	0.02933	0.00000	0.00000	0.00000	0.03843	0.01457	0.05300
0.00089	0.03080	0.00000	0.00000	0.00000	0.03169	0.00000	0.03080	0.02133	0.34861	0.02077	0.42151
0.00194	0.00000	0.01468	0.06898	0.00000	0.08560	0.00000	0.00000	0.00000	0.00000	0.00217	0.00217
0.00000	0.01478	0.00000	0.00000	0.00000	0.01478	0.00227	0.01478	0.02216	0.02546	0.01457	0.07924
0.00194	0.00000	0.01107	0.05046	0.02512	0.08859	0.00000	0.00000	0.00000	0.00000	0.00000	0.00000
0.00000	0.01984	0.00000	0.00000	0.00000	0.01984	0.00227	0.01984	0.02212	0.00694	0.01705	0.06822

**Table 11:** Calculation of NSPi, NSNi, ASi and Rank.

Sr. No.	NSPi	NSNi	ASi	Rank
1	0.93573	0.99961	0.96767	2
2	0.49010	0.80730	0.64870	9
3	1.00000	1.00000	1.00000	**1**
4	0.29012	0.78061	0.53537	13
5	0.41735	0.99446	0.70591	8
6	0.33673	0.85154	0.59414	10
7	0.47519	1.00000	0.73759	7
8	0.81302	0.87167	0.84234	4
9	0.25008	0.92706	0.58857	11
10	0.55931	0.92088	0.74009	6
11	0.22960	0.87426	0.55193	12
12	0.24804	0.00000	0.12402	16
13	0.67008	0.99485	0.83246	5
14	0.11566	0.81201	0.46384	15
15	0.69347	1.00000	0.84674	3
16	0.15527	0.83815	0.49671	14

**Table 12:** Validation experiments.

Method	Parametric Setting	Predicted Value						Experimental Value				
		AS	DA	Ra	Rz	WL	DR	DA	Ra	Rz	WL	DR
EDAS-PSO and EDAS	(A_on_)_8_(A_off_)_13_ (SV)_45_(WT)_8_	0.9137	95	3.163	22.996	0.0182	0.1277	95	3.125	22.755	0.0179	0.135
Trial Run	(A_on_)_8_(A_off_)_25_ (SV)_33_(WT)_8_	1	95.375	3.216	22.713	0.0286	0.1231	95.5	3.115	23.53	0.0108	0.125

## Data Availability

Available on request.

## Nomenclature

**Abbreviation**	**Description**
Aon, Ton	Pulse on-time
ANN	Artificial Neural Networks
Aoff, Toff	Pulse off-time
AS	Appraisal Score
BBD	Box-Behnken design
DA	Dimensional accuracy
df	Degree of freedom
DR	Diameter reduction
EDAS	Evaluation Based on Distance from Average Solution
m	number of alternatives
MS	Mean square
n	number of attributes
NDA	Negative distance from average
NSN	Normalized SN
NSP	Normalized SP
PDA	Positive distance from average
Pij	Performance value of the ith alternative corresponding to jth criterion
PSO	Particle Swarm Optimization
Ra	Average surface roughness
RSM	Response surface methodology
Rz	Root mean square surface roughness
SN	Sum of NDA
SP	Sum of PDA
SR	Surface roughness
SS	Sum of square
SV	Servo voltage
WEDM	Wire electric discharge machining
WF	Wire feed
WL	Wire loss
WT	Wire tension
